# Interactive influence of self and other language behaviors: Evidence from switching between bilingual production and comprehension

**DOI:** 10.1002/hbm.25044

**Published:** 2020-05-23

**Authors:** Huanhuan Liu, Chao Kong, Angela de Bruin, Junjie Wu, Yuying He

**Affiliations:** ^1^ Research Center of Brain and Cognitive Neuroscience Liaoning Normal University Dalian China; ^2^ Beijing Key Laboratory of Applied Experimental Psychology, Faculty of Psychology Beijing Normal University Beijing China; ^3^ Key Laboratory of Brain and Cognitive Neuroscience Liaoning Province Dalian China; ^4^ Department of Psychology University of York York UK; ^5^ State Key Laboratory of Cognitive Neuroscience and Learning & IDG/McGovern Institute for Brain Research, Beijing Normal University Beijing China

**Keywords:** attentional control, fMRI, language comprehension, language production, language switching

## Abstract

The neural mechanisms underlying one's own language production and the comprehension of language produced by other speakers in daily communication remain elusive. Here, we assessed how self‐language production and other‐language comprehension interact within a language switching context using event‐related functional Magnetic Resonance Imaging (er‐fMRI) in 32 unbalanced Chinese‐English bilinguals. We assessed within‐modality language interference during language production and comprehension as well as cross‐modality interference when switching from production to comprehension and vice versa. Results revealed that the overall effect of production (across switch and repeat trials) was larger in the cross‐modality than within‐modality condition in a series of attentional control areas, namely the left dorsolateral prefrontal cortex, anterior cingulate cortex and left precuneus. Furthermore, the left precuneus was recruited more strongly in switch trials compared to repeat trials (i.e., switching costs) in within‐production conditions but not in the cross‐modality condition. These findings suggest that switching from production to comprehension recruits cognitive control areas to successfully implement switches between modalities. However, cross‐language interference (in the form of language switching costs) mainly stems from the self‐language production system.

## Graphical abstract

The current study tested how self‐language production and other‐language comprehension interact within a language switching context. We found that other language behaviors affect our own language production via a series of attentional control areas, namely, the left dorsolateral prefrontal cortex, anterior cingulate cortex, and left precuneus, but cross‐language interference mainly stems from the self‐language system via the left precuneus.

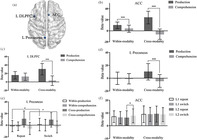

## 1 INTRODUCTION

Human communication requires an individual's self‐language system to interact with language input from others. Such interaction involves language production and language comprehension (Dijkstra & van Heuven, 1998, 2002; Grainger & Dijkstra, 1992; Grainger, Midgley, & Holcomb, 2010; van Heuven, Dijkstra, & Grainger, 1998; van Heuven, Schriefers, Dijkstra, & Hagoort, 2008). Therefore, communication is actually an alternate switching between production and comprehension. Compared to monolinguals, this flexible switching may be more effortful for bilinguals because of cross‐language interference due to the parallel activation of words from different languages (Dijkstra & van Heuven, 2002; Giezen, Blumenfeld, Shook, Marian, & Emmorey, 2015; Starreveld, De Groot, Rossmark, & Van Hell, 2014; van Heuven et al., 1998; van Heuven et al., 2008). An open question is how bilinguals ignore interference stemming from the speech of others (here referred to as “other‐language”) without affecting the normal operations of one's own language system (referred to as “self‐language”).

### 1.1 The neural basis of language control

In a conversation, bilinguals have to suppress not only other‐language interference, but also the disturbance from the self‐language system where two or more languages compete with each other. Within a bilingual's self‐language system, there can be interference from a nontarget language while switching from one language to the other. During this internal language switching, language control resources are recruited to suppress the interference from the nontarget language (Green, 1998). In particular, for unbalanced bilinguals, more language control will be required during L2 production to reduce L1 interference than vice versa. According to the IC hypothesis (Green, 1998), more inhibition of the L1 during L2 production should be observed than L2 inhibition during L1 naming.

A substantial body of studies on language control mechanisms during language production has identified the left dorsolateral prefrontal cortex (DLPFC) and anterior cingulate cortex (ACC) as primary centers of language control networks, which act jointly to handle competition, conflict, and interference control (Abutalebi & Green, 2016; Blanco‐Elorrieta & Pylkkänen, 2016, 2017; Blanco‐Elorrieta, Emmorey, & Pylkkanen, 2018; Branzi, Della Rosa, Canini, Costa, & Abutalebi, 2015; Chikazoe, Konishi, Asari, Jimura, & Miyashita, 2007; de Bruin, Roelofs, Dijkstra, & FitzPatrick, 2014; Luk, Green, Abutalebi, & Grady, 2012; Novick, Kan, Trueswell, & Thompson‐Schill, 2009; Novick, Trueswell, & Thompson‐Schill, 2005; Reverberi et al., 2018). Hernandez et al. (2000) and Hernandez (2009) compared a mixed‐language condition (with the target language switching between L1 and L2) and a single‐language condition (the target language is always the same language; Hernandez, 2009; Hernandez et al., 2000), and found more activation of the left DLPFC in the switching condition relative to the single‐language condition. These findings suggest that the DLPFC is required for response selection and inhibition of competing responses. Furthermore, Abutalebi et al. (2013) observed increased ACC activation in switching between languages, supporting the role of the ACC regarding monitoring of language selection (Abutalebi et al., 2013). The left DLPFC and ACC have also been implicated in cognitive control more generally (Chikazoe et al., 2007; Luk et al., 2012; MacDonald, Cohen, Stenger, & Carter, 2000; Novick et al., 2005).

These two regions might together be involved in bilingual language control, but are also linked to different phases of language production. Seo, Stocco, and Prat (2018) found that the ACC alone was significantly more active during the preparation of the target language than during the execution phase. In contrast, the left DLPFC and left IFG both showed significantly higher activation during the execution phase than during the target language preparing phase. However, previous studies studied production and comprehension separately, so it is unclear how the left DLPFC and ACC work in simultaneous production and comprehension. During daily‐life communication, this simultaneous production and comprehension involves switching between modalities to produce self‐language and comprehend language produced by others.

Furthermore, due to the within‐ and cross‐modality conditions involving self‐identification versus identifying others, we also focused on the left precuneus, which is engaged in attentional control and self‐representation. The precuneus plays a role in directing selective attention (Bischoff‐Grethe, Ivry, & Grafton, 2002; Dosenbach et al., 2007; Loose, Kaufmann, Auer, & Lange, 2003; Shomstein & Behrmann, 2006; Utevsky, Smith, & Huettel, 2014), and also plays a role in language switching (Guo, Liu, Misra, & Kroll, 2011; Reverberi et al., 2015; Reverberi et al., 2018). Reverberi et al. (2015) found that switch trials showed greater activation than nonswitch trials in the precuneus during the intention to speak phase. In addition, the precuneus might be involved in self‐representation and first‐person perspective of social interaction (Cavanna & Trimble, 2006; Farrer & Frith, 2002; Northoff & Bermpohl, 2004; Petrini, Piwek, Crabbe, Pollick, & Garrod, 2015). Petrini et al. (2015) found that the precuneus was more activated when viewing two agents interacting in atypical ways (socially incongruent conventions) rather than in typical ways (socially congruent conventions). The left precuneus in particular showed a more specific response to socially incongruent information. Accordingly, switching between people when going from production to comprehension or vice versa may cause interference to the self‐representation in joint language switching, that is, when participants need to identify whether they are speakers or listeners. Bilinguals might have to suppress/inhibit such interference in order to successfully switch between languages.

It has furthermore been suggested that our self‐language system might be affected by language input from interlocutors (Baus et al., 2014; Gambi & Hartsuiker, 2016; Kootstra, Hell, & Dijkstra, 2010). Blanco‐Elorrieta and Pylkkänen (2016) assessed how self‐production and self‐comprehension control mechanisms interact. Bilinguals were required to name target stimuli using different languages (i.e., language switching) in production, and to match verbal and visual stimuli printed in different languages by pressing buttons (i.e., category switching) in comprehension. Language‐switching in production involved the DLPFC while the left ACC was selectively activated during language‐switching comprehension, suggesting that language control in production differs from that in comprehension. Similar to this, Stasenko et al. (2020) also observed the involvement of control mechanisms in silently reading paragraphs (comprehension) and cued language switching (production). In the fMRI scanner, participants were required to silently read paragraphs written in just one language or paragraphs in which function or content words switched between languages. In a behavioral production task, participants named pictures in different languages according to cues. During silent reading, function word switches elicited costs in the bilateral DLPFC, and these neural switching costs were correlated with behavioral switching costs in the dominant language during the cued switching production task. The neural costs of silently reading language switches were similar to previous observations of switching costs in production (e.g., for review of this network see Abutalebi et al., 2008; Abutalebi & Green, 2016; Luk et al., 2012). This suggests that a modality‐general mechanisms underlies switching during silent reading and language production. However, most previous studies of language control focus on suppressing/inhibiting cross‐language interference within the bilinguals' self‐language system while interactive communication involves the bidirectional relationship between other‐language behavior and the self‐language system. Furthermore, control mechanisms might differ between production and comprehension. In the language production system, more top‐down control might be recruited, while in the language comprehension system more bottom‐up control of each language node might be involved (Dijkstra & van Heuven, 1998, 2002; Grainger et al., 2010; Grainger & Dijkstra, 1992; van Heuven et al., 1998, 2008), suggesting different control mechanisms are involved in production versus comprehension.

### 1.2 The current study

**FIGURE 1:**
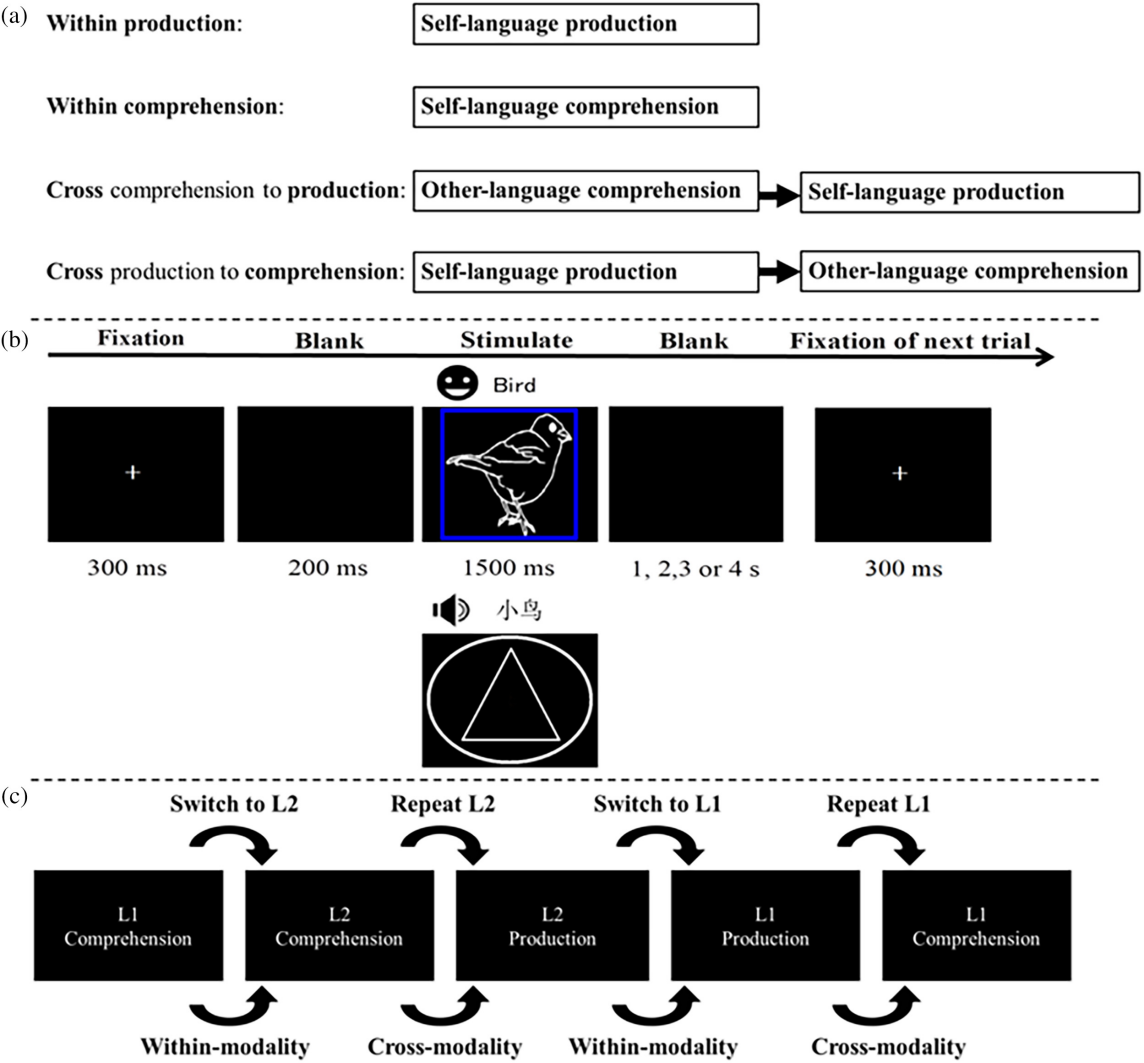
(a) Illustration of the self‐ and other‐language production and comprehension systems. Within‐production and comprehension separately represent self‐language production and comprehension systems. In addition, there were trials going from comprehension to production (hereafter: Cross‐modality production, indicating potential influence from other‐language comprehension on self‐language production) and trials going from production to comprehension (hereafter: Cross‐modality comprehension, indicating potential influence from self‐language production on other‐language comprehension). (b) Design and timing of the trial structure. Participants made a response following a cue. For example, a blue square around a bird picture meant “produce the picture name”, e.g., L2—“bird”, while a white circle around a triangle meant “listen to the word produced by another speaker”, for example, L1—“小鸟”. (c) Experimental conditions for joint language switching. The participant sometimes named the picture, while other times they listened to the recorded naming, thus forming two modalities of production and comprehension. The within‐modality condition was formed by two or more consecutive trials assigned to the same modality (speaking or listening). The cross‐modality condition consisted of trials requiring a switch from speaking to listening or vice. During repeat trials, the same language was used on two consecutive trials while switch trials required a switch between languages

The current study therefore used a language switching context, using within‐ and cross‐modality conditions to dissociate self cross‐language interference (within‐modality) from interference stemming from others (cross‐modality). We used the high spatial resolution of functional magnetic resonance imaging (fMRI) to identify the underlying bilingual control mechanisms when switching between bilingual production and comprehension. Such modality switching reflects the interactive influence between self and other language behaviors. As shown in Figure 1c, self‐language production versus comprehension of language produced by others are represented by within‐modality production and comprehension, respectively. The influence of other‐language production on self‐language production is measured by looking at cross‐modality production (producing a response yourself after having listened to another person's utterance), while the influence of self‐language production on comprehending other‐language is represented by cross‐modality comprehension (listening to another person's utterance after just having produced a response yourself). By comparing the bilingual control neural correlates between the within‐ and cross‐modality conditions, we can characterize how bilinguals resolve the interference stemming from self‐ and other‐language production and comprehension.

We hypothesized that if cross‐language interference during self‐language production is affected by other‐language information or vice versa, switching costs should differ between the cross‐modality and within‐modality conditions. In that case, the Modality sequence (Within, Cross) should interact with the Language sequence (Repeat, Switch). Higher switch costs for cross‐ than within‐modality in the language‐control ROIs (left DLPFC, ACC, left precuneus) would suggest that other‐language production elicit greater cross‐language interference than self‐production. In contrast, larger switch costs within‐modality than cross‐modality would suggest that cross‐language interference is mainly generated by the self‐language system. Altogether, this study aimed to shed light on how self‐ and other‐language systems interact in everyday communication by assessing language‐control mechanisms while switching between production and comprehension.

## 2 METHODS

### 2.1 Participants

Thirty‐nine unbalanced Chinese‐English bilingual students studying at Liaoning Normal University participated in this study. All participants were native speakers of Chinese with an intermediate knowledge of English (see Table 1). Chinese is the dominant language in their family‐ and school‐life, they learned English in class and communicate in English in some situations. Apart from English, they did not have exposure to other foreign languages, so English can be regarded as L2. All were right‐handed and they all had corrected‐to‐normal visions or normal sights. Participants with a history of neurological or psychiatric conditions, or use of psychotropic medication were excluded. All procedures in the present study were authorized by the Ethics Committee of Research Center of Brain and Cognitive Neuroscience of Liaoning Normal University. Data from seven participants were deleted because of excessive in‐scanner head motion. The final simple data consisted of 32 participants (9 males), aged from 18 to 25 (M = 22.3 ± 2.0 years).

**TABLE 1:** Participants' language characteristics showing means with standard deviations in parentheses

Language profile	L1 (Chinese)	L2 (English)
*Self‐rating*		
AoA	‐	12.60 (2.56)
Listening	5.53 (.62)	3.44 (.76)
Speaking	4.97 (.47)	3.25 (0.80)
Reading	4.34 (1.10)	2.72 (1.14)
Writing	4.91 (.86)	3.19 (1.09)
*Proficiency test*		
OPT	‐	36.25 (4.00)

Table 1 shows the age of L2 acquisition (AoA), self‐rated language skills (including Listening, Speaking, Reading, and Writing), and the results from the Oxford Placement Test (OPT). The self‐rating questionnaire was used to obtain subjective proficiency. Participants indicated the proficiency of their L1 and L2 Listening, Speaking, Reading, and Writing skills using a six‐point scale in which 6 indicated that L1/L2 knowledge was perfect, and 1 indicated no knowledge of L1/L2. Results of paired‐samples t‐tests showed significant differences of language skills between L1 and L2 in Listening (L1: 5.53 ± .62, L2: 3.44 ± .76, *t*(31) = 12.758, *p* < .001), Speaking (L1: 4.97 ± .47, L2: 3.25 ± .80, *t*(31) = 11.965, *p* < .001), Reading (L1: 4.34 ± 1.10, L2: 2.72 ± 1.14, *t*(31) = 9.423, *p* < .001), and Writing (L1: 4.91 ± .86, L2: 3.19 ± 1.09, *t*(31) = 6.85, *p* < .001). OPT is a validated placement test published by Oxford University Press (Allan, 2004). Due to time restrictions, we adopted an abbreviated measure that randomly extracted 25 multiple choice questions and a cloze test with 25 questions (see Appendix A). The total score was of 50 points. The average scores of 36 points on the OPT were analogous to previous studies of Chinese—English unbalanced bilinguals with intermediate L2 proficiency (Liang & Chen, 2014; Liu, Liang, Dunlap, Fan, & Chen, 2016).

### 2.2 Joint language switching materials

We selected 48 black‐and‐white line drawings from the Snodgrass and Vanderwart's photo gallery as language switching stimuli, which were standardized by Zhang and Yang (2003). Each Chinese name consisted of two characters, and their English name consisted of one or two syllables with three to six letters. Forty bilinguals who did not participate in this experiment rated the subjective familiarity of Chinese and English words on a 5‐point scale (1 = “very unfamiliar”, 5 = “very familiar”). There were no significant differences between subjective Chinese name familiarity and subjective English name familiarity (L1: 4.79 ± .12, L2: 4.81 ± .10, *t*(47) = −1.48, *p* > .05) nor between Chinese word frequency and English word frequency (L1: 77.53 ± 114.24, L2: 104.23 ± 128.39, *t*(47) = 1.54, *p* > .05; Chinese word frequency: Cai & Brysbaert, 2010; English word frequency: Brysbaert & New, 2009). None of the stimuli were Chinese‐English cognates (see Appendix B). The Chinese and English comprehension trials were recorded by a professional female speaker whose proficiency in the two languages is balanced with a native‐like Mandarin and English pronunciation.

### 2.3 Procedure

The experiment used a 2 (Modality: Production vs. Comprehension) × 2 (Modality sequence: Repeat vs. Switch) × 2 (Language: L1 vs. L2) × 2 (Language sequence: Repeat vs. Switch) design. Since the naming latencies were not captured during fMRI, the experiment included a separate fMRI and behavioral part. The behavioral part was the same as the fMRI part, completed at least 7 days later to avoid repetition priming and a reduction in switch costs as a result of practice. The fMRI part was a 1‐hr language switching task in the fMRI scanner, divided into 6 scan runs. Each run contained 98 trials, including 2 warm‐up trials and 96 experimental trials, lasting 7 min and 40 s. The 96 trials were evenly distributed to 16 conditions with 6 trials in each condition. Trials were pseudo‐randomly ordered, and there were no more than 3 consecutive trials of the same condition. Overall, there were 576 formal trials and 12 warm‐up trials which resulted in 36 trials per condition. The language switching task was programmed using the psychology software E‐prime 2.0. Before the formal experiment, participants were familiarized with all materials in both languages and practiced the switching task. This practice language switching task followed the same procedure as the actual experiment and included 24 trials with pictures that were not used in the experiment.

As shown in Figure 1b, each trial started with a white fixation at the center of the black‐background screen for 300 ms, followed by a blank screen lasting 200 ms, and then followed by a line drawing presented with a colored cue (production task) or a cue (comprehension task) for 1,500 ms. The visual (production) and auditory (comprehension) stimuli were always presented separately. A cue instructed participants which language to use to name the picture in the production task. If the cue was a red or blue square, it required participants to name the picture in L1 or L2. In comprehension trials, a meaningless picture (i.e., a white triangle surrounding a white circle)^1^ was presented to match the visual cue in production trials and to instruct participants to pay attention to the audio stimulus. Finally, a blank screen lasted 1, 2, 3, or 4 s. In order to simulate language comprehension in real life, participants just listened passively and did not respond to the comprehension trials. To remind participants to listen carefully to the words heard in the comprehension trials during the fMRI scanning, we asked them at the end of each session to judge whether a word had been heard during the task. This recognition task visually presented participants with a Chinese or English word and asked them to indicate whether they had heard the word during the task. Each recognition task included 12 trials per run. Six words had been heard in the preceding run and another six had not been heard. These words were not counterbalanced for L1 and L2. If participants thought they had heard the word during the task, they had to press “1”, otherwise they had to press “2”. The average accuracy of the recognition task was 61%, which was significantly different from guessing rate (Range = 53–69, *M* = 60.59 ± 3.63% > 50%, *t*(31) = 16.52, *p* < .001). The results from the recognition task were not analyzed further as this task was just used to encourage participants to pay attention to the comprehension trials. The behavioral experiment was similar to the fMRI experiment and naming latencies were recorded by PSTSR‐BOX.

### 2.4 fMRI data acquisition

Functional MRI data were collected with a GE Discovery MR750 3T scanner. Participants were instructed to keep their heads still during scanning. Functional images were collected using a T2*‐weighted EPI sequence. Volumes covered the whole brain (repetition time = 2000 ms, echo time = 30 ms, flip angle = 90°, sequential acquisition = 33 axial slices, slice thickness = 3.5 mm, image matrix = 64 × 64, field of view = 224 × 224 mm, voxel size = 3.5 × 3.5 × 4.2 mm). Each functional scanning session contained 225 time points, with a total of 6 runs. Structural images were collected using a T1‐weighted 3‐D MPRAGE sequence (repetition time = 6.652 ms, echo time = 2.928 ms, flip angle = 12°, sequential acquisition = 192 slices, slice thickness = 1 mm, spacing between slices = 1 mm, image matrix = 256 × 256, field of view = 256 × 256 mm, voxel size = 1 × 1 × 1 mm), in order to coregister with the functional images.

### 2.5 fMRI data preprocessing

fMRI data were preprocessed by DPABI (DPABI: a toolbox for Data Processing & Analysis for Brain Imaging (Yan, Wang, Zuo, & Zang, 2016). In the first step, all the EPI DICOM data were converted to NIFTI format. The first 5 volumes of each run were discarded because of T1 relaxation artifacts. Second, all volumes slice scan times were corrected to the middle time slice and realigned to the first scan to correct for head motion. Third, the structural images of each subject were coregistered with the mean functional images and then images were normalized to the Montreal Neurological Institute template. Fourth, all voxels were resampled to 3 × 3 × 3 mm. Last, all functional volumes were smoothed by using a 6‐mm FWHM isotropic Gaussian kernel.

### 2.6 fMRI data statistical analysis

The fMRI data were analyzed by using SPM12 (Wellcome Department of Cognitive Neurology, London, UK), based on MATLAB R2013b. We excluded the data of 7 participants because of excessive in‐scanner head motion (3 mm, 3°). Data of 32 participants were analyzed. In the first level, the fMRI data was performed by the general linear model (GLM). We modeled the onset times of each trial in each of the session, there were 16 types of trials totally. Six head motion parameters of each session per participant were also modeled as noise regressors. These vectors were convolved with the canonical hemodynamic response function.

### 2.7 ROI analysis

**FIGURE 2:**
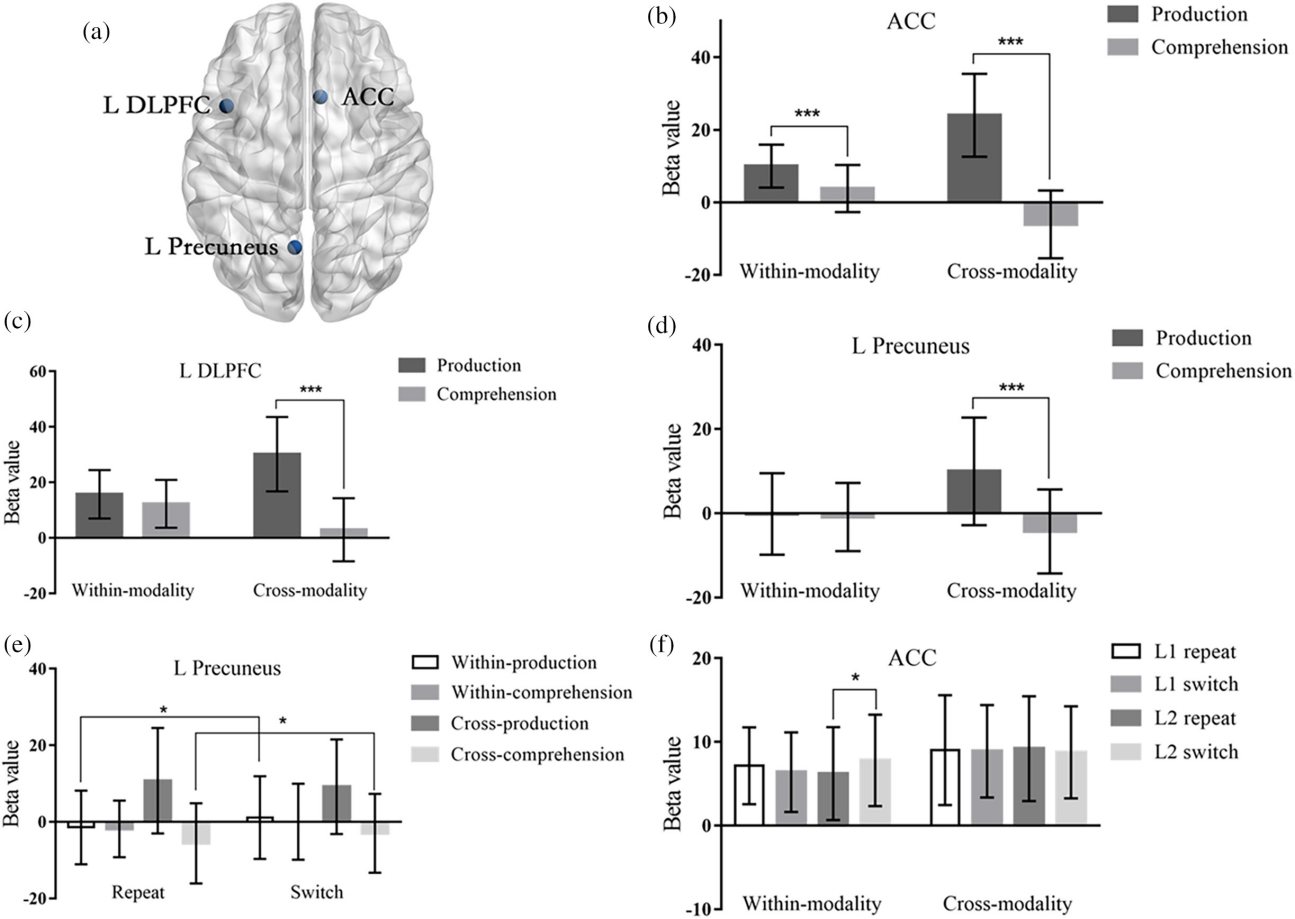
Further analysis of the interactions. (a) The image shows the included ROIs (ACC, left DLPFC, left Precuneus). (b–d) The graphs show the contrast estimates for Modality (Production, comprehension) by Modality sequence (Within‐modality condition, Cross‐modality condition). (e) The graphs show the contrast estimates for Modality (Production, comprehension) by Modality sequence (Within‐modality condition, Cross‐modality condition) and by Language sequence (Repeat, Switch). (f) The graphs show the contrast estimates for Modality sequence (Within‐modality condition, Cross‐modality condition) by Language (L1, L2) and by Language sequence (Repeat, Switch). Error bars show the standard error of the mean. The asterisks indicate the significant pairwise differences between the corresponding conditions

For the ROI analysis, we selected the following coordinates according to previous neuroimaging studies (see Table 2 and Figure 2a). The centroid MNI coordinates were used as centers of spherical ROIs and all radius were 6 mm. Mean beta values within each ROI were calculated for each combination of the four conditions, yielding 16 different combinations totally. To further illuminate the interactive influence of self‐ and other‐language behavior on our own language production and comprehension system, data for each ROI were respectively submitted to planned four‐way repeated‐measures ANOVAs crossing Modality (Production, Comprehension) × Modality sequence (Within‐modality, Cross‐modality) × Language (L1, L2) × Language sequence (Repeat, Switch), using Statistical Product and Service Solutions (SPSS) 18.0. We mainly focused on the interaction of Modality, Modality sequence and Language sequence which reflects the influence of switching between two modalities on language control. Nonsignificant main effects and interactions are not reported.

**TABLE 2:** Description of regions of interest (ROIs) used for neuroimaging analysis

Region	Reference	Original coordinates	Centroid MNI coordinates	Radius (mm)
L DLPFC	Luk et al. (2012)(Talairach coordinates)	−46, 18, 26	−44, 13, 29	6
ACC	Guo et al. (2011)(MNI coordinates)	7, 18, 41	7, 18, 41	6
L Precuneus	Sperduti, Delaveau, Fossati, and Nadel (2011)(MNI coordinates)	−7, −64, 50	−7, −64, 50	6

Abbreviations: L, left; R, right.

## 3 RESULTS

### 3.1 Naming behavioral results

Since participants only listened passively, there were no behavioral data for comprehension trials. Modality could therefore not be included as a variable in the behavioral data analysis. Due to the high naming accuracy in the language production task (> 95%), we only analyzed naming latencies. We removed the data from the first two trials in each block as well as the data from naming latencies beyond *M ±* 3*SD* (8.30%) and from incorrect responses. For naming latencies, a three‐way repeated‐measures ANOVA was run with the three within‐subject factors: Modality sequence (Within‐modality, Cross‐modality) × Language (L1, L2) × Language sequence (Repeat, Switch). Nonsignificant main effects and interactions are not reported.

As shown in Table 3, we found main effects of (a) Modality sequence, where participants named more slowly in the Cross‐modality trials (877 ± 143 ms) relative to the Within‐modality trials (841 ± 137 ms), (b) Language, where participants named more slowly in L1 (888 ± 148 ms) compared to L2 (829 ± 133 ms), (c) Language sequence, where responses were slower in Switch trials (868 ± 146 ms) compared to Repeat trials (849 ± 135 ms). Additionally, a significant interaction was found between Modality sequence × Language sequence. In the Within‐modality condition (production–production), Switch trials (861 ± 151 ms) were significantly slower than Repeat trials (821 ± 127 ms) (*p* < .001), while such difference in the Cross‐modality condition (comprehension–production) was not significant (875 ± 143 ms for Switch trials, 878 ± 146 ms for Repeat trials, *p* = .68).

**TABLE 3:** Main and interaction effects of naming latencies

Effects	Comparisons	*F*	*p*	*η* ^*2*^
Modality sequence	Cross > Within	32.89	<.001	.52
Language	L1 > L2	61.78	<.001	.67
Language sequence	Switch > Repeat	8.93	.005	.22
Modality sequence × Language sequence	—	18.27	<.001	.37

### 3.2 ROI results

As shown in Table 4, there were significant main effects of Modality, Modality sequence, Language, and Language sequence.

**TABLE 4:** Main effects on regions of interest

Main effects	ROIs	Comparisons	*F*	*p*	*η* ^*2*^
Modality	L DLPFC	Production > Comprehension	62.89	<.001	.67
	ACC	Production > Comprehension	99.87	<.001	.76
	L Precuneus	Production > Comprehension	13.49	.001	.3
Modality sequence	L DLPFC	Cross > Within	18.36	<.001	.37
	ACC	Cross > Within	15.1	.001	.33
	L Precuneus	Cross > Within	30.95	<.001	.50
Language	L Precuneus	L1 > L2	4.29	.047	.12
Language sequence	L Precuneus	Switch > Repeat	5.48	.026	.15

Abbreviations: L, left; R, right.

#### 3.2.1 Modality effect

There was more activation in the Production condition relative to the Comprehension condition in the left DLPFC, ACC, and Left Precuneus.

#### 3.2.2 Modality sequence effect

The Cross‐modality condition recruited the left DLPFC, ACC, and left Precuneus more strongly compared to the Within‐modality condition.

#### 3.2.3 Language effect

Using L1 elicited greater activation of left Precuneus relative to using L2.

#### 3.2.4 Language sequence

Increased activation in Switch trials compared to Repeat trials was obtained in the left Precuneus.

As shown in Tables 5 and 6, there were interaction effects and follow‐up analyses, respectively.

**TABLE 5:** Interaction effects on regions of interest

Interaction effects	ROIs	*F*	*p*	*η* ^*2*^
Modality × Modality sequence	L DLPFC	45.2	<.001	.59
	ACC	52.7	<.001	.63
	L Precuneus	27.95	<.001	.47
Modality sequence × Language	L Precuneus	8.91	.005	.22
Modality × Modality sequence × Language sequence	L Precuneus	4.98	.033	.14
Modality sequence × Language × Language sequence	ACC	6.15	.019	.17

Abbreviations: L, left; R, right.

**TABLE 6:** Follow‐up analysis on interaction effects

Interaction effects	ROIs	Comparisons	*F*	*p*	*η* ^*2*^
Modality × Modality sequence	L DLPFC	Within: Production > Comprehension	3.70	.063	.11
		**Cross: Production > comprehension**	69.94	< .001	.69
		**Production: Cross > Within**	61.38	< .001	.66
		**Comprehension: Within > Cross**	24.39	< .001	.44
	ACC	**Within: Production > Comprehension**	17.01	< .001	.35
		**Cross: Production > Comprehension**	92.89	< .001	.75
		**Production: Cross > Within**	61.58	< .001	.67
		**Comprehension: Within > Cross**	34.84	< .001	.53
	L Precuneus	Within: Production > Comprehension	.16	.693	.01
		**Cross: Production > Comprehension**	24.69	< .001	.44
		**Production: Cross > Within**	42.63	< .001	.58
		**Comprehension: Within > Cross**	7.23	.011	.19
Modality sequence × Language	L Precuneus	**L1: Cross > Within**	39.24	< .001	.56
		**L2: Cross > Within**	4.34	.045	.12
		Within‐modality: L2 > L1	.96	.335	.03
		**Cross‐modality: L1 > L2**	10.21	.003	.25
Modality × Modality sequence × Language sequence	L Precuneus	**Within‐production: Switch > Repeat**	4.81	.036	.13
		Cross‐production: Repeat > Switch	2.33	.137	.07
		Within‐comprehension: Switch > repeat	2.53	.122	.08
		**Cross‐comprehension: Switch > repeat**	6.36	.017	.17
Modality sequence × Language × Language sequence	ACC	L1 within‐modality: Repeat > Switch	1.40	.245	.04
		**L2 within‐modality: Switch > Repeat**	6.85	.014	.18
		L1 cross‐modality: Repeat > Switch	.08	.786	< .001
		L2 cross‐modality: Repeat > Switch	.04	.560	.01

*Note*: Significant results are marked bold. Although follow‐up analyses showed significant comprehension switch costs in the cross‐comprehension condition but not in the within‐comprehension condition, the interaction between Language sequence × Modality sequence is only significant for production but not for comprehension (see further analysis in *g*) *Modality* × *Modality sequence* × *Language sequence effect*). That is, the effect of language sequences differs between within‐modality and cross‐modality for production trials but not for comprehension trials.

Abbreviations: L, left; R, right.

#### 3.2.5 Modality × Modality sequence effect

There was an interaction between Modality × Modality sequence in the left DLPFC, ACC, and left Precuneus (see Figure 2b–d). Overall, activation was larger for production than comprehension trials, but this was especially the case on cross‐modality trials. As shown in Table 6, in all three ROIs, the production condition showed increased activation for cross‐modality than within‐modality trials. In other words, when a production trial was preceded by a comprehension trial, activity in the left DLPFC, ACC, and left Precuneus increased as compared to production being preceded by production. The opposite was observed for comprehension: Activation in all three ROIs increased when the previous trial was a comprehension trial too as compared to the preceding trial requiring production.

#### 3.2.6 Modality sequence × Language effect

There was an interaction between Modality sequence × Language in the left Precuneus. Overall, cross‐modality trials showed increased activation compared to within‐modality trials, but this was more strongly the case for L1 trials. As shown in Table 6, the cross‐modality condition showed increased activation for the L1 than L2 while no significant difference between languages was found in the within‐modality condition.

#### 3.2.7 Modality × Modality sequence × Language sequence effect

Of main interest was the three‐way interaction of Modality × Modality sequence × Language sequence, which reached significance in the left Precuneus. This suggests that effects of modality sequence on switching costs (i.e., language sequence) differed between the production and comprehension modality. We therefore ran a follow‐up 2 × 2 analysis looking at the interaction between Modality sequence and Language sequence in Production and Comprehension separately. The interaction between Modality sequence and Language sequence was significant in Production (*F*_(1,31)_ = 6.20, *p* = .018, *η* ^*2*^ = .17). As can be seen in Table 6, there was a significant switching cost within‐modality (i.e., when production was preceded by production). In contrast, when production was preceded by comprehension, there was no switching cost. The interaction between Modality sequence and Language sequence was not significant in the comprehension condition (*F*_(1,31)_ = .21, *p* = .65, *η* ^*2*^ = .01). As can be seen in Table 5, there was only a significant switching cost when comprehension was preceded by production (i.e., cross‐modality) while this switching cost did not reach significance when comprehension was preceded by comprehension (i.e., within‐modality). Nevertheless, there was no significant interaction between Modality sequence and Language sequence in comprehension, suggesting that switching costs were comparable during comprehension, regardless whether the previous trial was production or comprehension. To confirm this, we directly compared the switching cost (the beta value of switch trials minus the repeat trials) between Within‐modality and Cross‐modality during production and comprehension, respectively. A one‐way repeated‐measure ANOVA revealed that in Production, the switching cost in Within‐modality condition was higher than in Cross‐modality (*F*_(1,31)_ = 6.20, *p* = .018, *η* ^*2*^ = .17). During comprehension, the switching cost in Cross‐modality did not differ from the Within‐modality (*F*_(1,31)_ = .21, *p* = .647, *η* ^*2*^ = .01).

#### 3.2.8 Modality sequence × Language × Language sequence effect

The three‐way interaction of Modality × Modality sequence × Language sequence yielded significance in the ACC. As shown in Table 5, greater activation in L2 Switch trials than in L2 Repeat trials was observed in the Within‐modality condition but not in any of the other conditions (see Figure 2f).

To summarize, first, production recruited more language control resources from the ACC, left DLPFC and left precuneus compared to comprehension across modalities, suggesting that language control in production is more difficult than control in comprehension. Across switch and repeat trials, this difference between production and comprehension was larger in the cross‐modality condition than in the within‐modality condition. This suggests that, overall, self‐language production especially recruited additional resources when preceded by comprehension of language produced by others (see Figure 2b–d).

Second, an overall switching cost was observed. In the ACC, this switching cost was most clearly present in the L2 in the within‐modality condition. Of main importance for this study, the switching cost (across L1 and L2) was modulated by modality sequence and modality in the left precuneus. During production, a significant switching cost was only observed in the within‐modality condition while no significant switching cost was found in the cross‐modality condition (if anything, numerically, the switching cost went in the opposite direction). During comprehension, the switching cost was not significantly affected by the previous trial being of the same or the other modality.

## 4 DISCUSSION

By studying how self‐ and other‐language production and comprehension interact with each other, this work identified cross‐modality effects during both production and comprehension, but in different ways. Overall, production recruited the left DLPFC, ACC, and left precuneus more strongly than comprehension, and this difference was largest when production was preceded by comprehension. Furthermore, switching costs (behaviourally and in terms of activation in the left preceneus) during language production were only observed within‐modality but not when preceded by comprehension of language produced by others (cross‐modality). Overall, the findings show differences in language/cognitive control mechanisms recruited during production and comprehension as well as differential effects when going from one modality to the other as compared to staying in the same modality. These ideas are developed as follows.

### 4.1 Switching between production and comprehension reflects a combination of stimulus, response, and identity modalities

The current study involved switching between production and comprehension in a way that combines switching between stimulus modalities (visual vs. auditory input) and switching between response modalities (speaking vs. listening). We found that, overall, production was more effortful than comprehension, especially in the cross‐modal condition. The ACC might have been recruited for conflict monitoring of the modality switch, and the left DLPFC and the left precuneus may have blocked cross‐modality interference. This suggests that switching between stimulus response modalities might be similar to task switching and might recruit similar cognitive control areas (Bischoff‐Grethe et al., 2002; Chikazoe et al., 2007; Dosenbach et al., 2007; Loose et al., 2003; Luk et al., 2012; MacDonald et al., 2000; Novick et al., 2005; Shomstein & Behrmann, 2006; Utevsky et al., 2014). This might be because selecting and executing a goal‐directed response to a given stimulus creates a representation which integrates—or “binds”—features such as, stimulus attributes and the corresponding response (e.g., Henson, Eckstein, Waszak, Frings, & Horner, 2014; Waszak, Hommel, & Allport, 2003). This Stimulus–Response binding is easier to process in the same modality (i.e., within modality). However, a preceding S‐R event‐file (i.e., trial *n* − 1) interferes with a subsequent S‐R event‐file (i.e., trial *n*) when moving from one modality to another, because their integrated features (stimulus—task—response properties “bound together”) are incompatible. At this time, it is necessary to reactivate/retrieve the new S‐R binding and/or inhibit the previous S‐R binding. Feature binding and response retrieval operate in separation and are independently modulated by top‐down (e.g, attentional weighting) and bottom‐up (e.g., encoding and retrieving regulation) influences (Frings et al., 2020). Thus, unbalanced bilinguals might recruit cognitive control areas such as the left DLPFC and ACC (Chikazoe et al., 2007; Luk et al., 2012; MacDonald et al., 2000; Novick et al., 2005) and left precuneus (Bischoff‐Grethe et al., 2002; Dosenbach et al., 2007; Loose et al., 2003; Shomstein & Behrmann, 2006; Utevsky et al., 2014) to bind and retrieve S‐R features during modality switching.

In addition, modality switching also involves identity switching (i.e., switching between yourself and the interlocutor). The left precuneus is linked to self‐representation and first‐person perspectives during social interaction (Cavanna & Trimble, 2006; Farrer & Frith, 2002; Northoff & Bermpohl, 2004; Petrini et al., 2015). According to the account of Stimulus–Response binding (e.g., Henson et al., 2014; Waszak et al., 2003), participants likely formed an identity of the other speaker at the beginning of the experiment. Increased activation in the left precuneus across switch and repeat trials during cross‐modality production compared to within‐modality production could reflect increased effort to switch away from listener identity in the previous trial to speaker identity in the current trial. In addition to the left precuneus, the left DLPFC and ACC were also activated, indicating that identity set switching potentially induced a conflict to be solved.

Taken together, switching between production and comprehension is likely to include different processes, including switches between stimulus modalities (visual vs. auditory input), between response modalities (speaking vs. listening), and between identity modalities (speaker vs. listener). The increased activation in general cognitive control areas during cross‐modality trials compared to within‐modality trials might reflect a stimulus modality switch independent of a switch in the linguistic task itself. However, these three processes together are also needed during communication in real life, as conversations include switches between different sorts of input, different types of responses, and different people.

### 4.2 Cross‐language interference primarily stems from the self‐language production system

While production overall recruited more control areas when switching from comprehension to production, effects of cross‐language interference were observed within‐modality during production trials. Language switching costs were larger when switching from one language to the other within production trials than when switching from comprehension to production.

The Bilingual Interactive‐Activation Model from a developmental perspective (BIA‐d; Grainger et al., 2010) points out that the switching cost in language comprehension relies on exogenous control because words passively activate the corresponding language node via bottom‐up activation, which then inhibits activity in the other language's lexical processes. During language production, switching actively employs endogenous control because the intention to produce Language A activates the corresponding language node via top‐down activation, which results in inhibiting the activity of the other language. Hence, due to different control mechanisms, the amount and type of control recruitment may be different between language production and comprehension. Overall, production recruited language control areas (left DLPFC, ACC, and left precuneus) more strongly than comprehension trials did. In terms of cross‐language interference, we found that, in the within‐production condition, switch trials showed greater activation than repeat trials in the left precuneus, consistent with an attentional control role in suppressing/inhibiting the previous task set and directing attention to the current one (Guo et al., 2011; Reverberi et al., 2015, 2018). This pattern was not observed in the cross‐production condition. The fMRI data were in line with the behavioral data showing switch costs in the within‐production condition but not in the cross‐production condition. Such cross‐language interference seems to stem from the self‐language production system, which is governed by endogenous control mechanisms.

In comprehension, the switching cost does not significantly differ between the previous trial being production versus the previous trial being comprehension. Furthermore, comprehension recruited the left DLPFC, ACC, and left precuneus less strongly than production. Comprehension requires processes from the lexical to semantic level while production requires the opposite. For unbalanced bilinguals, it might be easier to get the meaning of a word compared to producing a word. In other words, the bottom‐up activation via exogenous control might be less effortful than the top‐down activation via endogenous control. These perceptual processes might have been unaffected by language production considering that we found similar switch costs in within‐ and cross‐modality comprehension. Our findings were in line with a behavioral study of Declerck, Koch, Duñabeitia, Grainger, and Stephan (2019). They did not show switching costs during comprehension‐based language switching tasks even though task‐switch costs, modality‐switch costs, and production‐based language switch costs were found.

Our study reaches a different conclusion than some behavioral work that has suggested that language production and comprehension (partially) share control mechanisms (Peeters, Runnqvist, Bertrand, & Grainger, 2014). In this study, participants had to switch between naming pictures and making language judgments or semantic classifications on words preceding the pictures. Participants were slower to name the picture in their L1 after reading a word in their L2 (but not in their L2 after reading in L1), showing that switching costs can occur when switching from comprehension to production. Furthermore, our results are different from Stasenko et al.'s (2020) conclusion that language production and comprehension might share a general switching mechanism. However, recent behavioral and neuroimaging studies have reached similar conclusions to ours, namely, that different mechanisms might underly comprehension and production of switches. Blanco‐Elorrieta and Pylkkänen (2016) revealed that language control in language production recruited the DLPFC during language switching, and that language comprehension employed the left ACC during category switching. Furthermore, Blanco‐Elorrieta and Pylkkänen (2017) also found dissociations between language switching in language production and comprehension. During language production, switching costs in the DLPFC and ACC varied depending on the context (bilingual cues, monolingual cues, or artificial cues). During comprehension, the switch effect in the dlPFC and ACC was similar across the three contexts.

Our findings suggest that production and comprehension differ in the degree of top‐down versus bottom‐up mechanisms. Another possibility is that when you have only passively listened to a language, there is not enough interference to cause a switching cost when you have to use another language on the next trial. As a consequence, there might not have been a switching cost when going from comprehension to production (i.e., cross‐production condition). In contrast, within‐modality production requires active naming, in which case the language schema used in the previous and current trial might conflict (Branzi et al. (2015); de Bruin et al., 2014; Green, 1998; Guo et al., 2011; Reverberi et al., 2015; Reverberi et al., 2018; Seo et al., 2018). Therefore, such conflict should be solved or suppressed, recruiting the left precuneus more strongly. The finding that a larger switch cost was induced in within‐modality production compared to cross‐modality production confirms this speculation. This suggest that cross‐language interference primarily stems from the self‐language production system.

Taken together, a significant switching cost in the left precuneus was observed during the within‐modality production condition but not in the cross‐modality condition, indicating that cross‐language interference comes from the self‐language system. These findings are in line with daily‐life experiences: we can focus on a conversation in a loud room by suppressing/inhibiting other voices. Somehow, even with large amounts of information flooding our senses, we are able to focus on what is important. Therefore, attentional control areas might be needed to sequence these processes in the correct order to make sure that we are not distracted by information stemming from others as well as cross‐language interference stemming from our production.

### 4.3 Limitations

Although our study is a first attempt to reveal the neural mechanisms involved in managing one's own language production and comprehension, there are some limitations. First, the noisy fMRI environment did not allow us to record overt naming during scanning. In line with previous language switching studies (e.g., Abutalebi et al., 2008; Guo et al., 2011; Wu et al., 2019), participants completed the fMRI experiment first, followed by a behavioral session in which overt naming was recorded. The order of the fMRI and behavioral sessions was not counterbalanced. Potential practice effects, including increased familiarity with the stimuli and words, may be the reason why no interaction between language and other variables was observed in the behavioral data. However, we still found the interaction of interest (i.e., between modality sequence and language sequence), in line with the fMRI results. That is, the switch costs were significant in the within‐production condition but absent in the cross‐production condition. We hope to improve the recording of fMRI voice response in the future. Second, to simulate real‐life language comprehension, the comprehension task did not require an explicit response. Hence, the behavioral data lack comprehension data, and cannot be analyzed in the same way as the fMRI data. The fMRI results show some differences between production and comprehension that could not be examined in the behavioral data. For example, the fMRI results found greater activation of the left precuneus during L1 trials relative to L2 trials across both production and comprehension. This is in line with the behavioral production data showing slower L1 than L2 naming responses during language production. It suggests an increased need to release the increased inhibition applied to the dominant L1 (strong inhibition required since it is the dominant language) as compared to L2 (weak inhibition required since it is the weaker language; e.g., Costa & Santesteban, 2004; Costa, Santesteban, & Ivanova, 2006; Declerck & Philipp, 2015; Li, Liu, Pérez, & Xie, 2018; Meuter & Allport, 1999; Philipp, Gade, & Koch, 2007; Philipp & Koch, 2009). In the absence of behavioral comprehension data, however, it remains an open question whether L1 comprehension also takes longer than L2 comprehension. Finally, it should be noted that the observed interactions resulted in a large number of posthoc tests. Our conclusions will need further support in future studies.

## 5 CONCLUSION

The current study created a bilingual context in which bilinguals had to alternate between producing and comprehending two languages. Two main findings were observed. The left DLPFC, ACC, and left precuneus were activated most strongly during language production trials when moving from comprehension to production, suggesting that comprehension of other language behaviors affects subsequent self‐language production. When looking at cross‐language interference, however, switching costs during language production were only found within the production modality but not when moving from comprehension to production. Together these data suggest that increased attentional control is needed during language production when switching between modalities but that interference between languages mainly stems from self‐language production.

## CONFLICT OF INTEREST

No potential conflict of interest was reported by the authors.

## DATA AVAILABILITY STATEMENT

The data used to support the findings of this study are available from the corresponding author upon request.

## Endnote

^1^ According to the Bilingual Interactive Activation model (BIA; Grainger & Dijkstra, 1992; van Heuven et al., 1998) and its developmental variant (BIA‐d; Grainger et al., 2010), comprehension‐based language control starts with the activation of a word representation (e.g., “bird”) that automatically activates its corresponding language node, which is a mental representation of language membership. Thus, comprehension trials did not have cues to provide such language membership. To enhance comparability between comprehension and production trials, the language cue was presented simultaneously with the picture in the production task. The fMRI data therefore reflect the integration of language selection and language production processes rather than language selection preparation in response to the cue.

## Appendix A. OXFORD PLACEMENT TEST

**Instruction 1:** Please tick the corresponding number you think is the correct answer.

**Table:**

**1**	Water _________ at a temperature of 0°C.
	1、be freezing；2、freezes；3、 is freezing.
**2**	In some countries ___ dark all the time in winter.
	1、 there is； 2、 is； 3、it is
**3**	In some countries people wear light clothes_____ cool.
	1、for keeping；2、to keep；3、for to keep.
**4**	In Madeira they have _____ weather almost all year.
	1、the good; 2、good; 3、a good.
**5**	Most Mediterranean countries are______ in October than in April.
	1、more warm; 2、the more warm; 3、 warmer.
**6**	Parts of Australia don't have _______ rain for long periods.
	1、the; 2、some; C、any.
**7**	In the Arctic and Antarctic _____ a lot of snow.
	1、it is; 2、there is; 3、it has.
**8**	Climate is very important in ____ people's lives.
	1、 most; 2、of most; 3、the most.
**9**	Even now there is ____ we can do to control the weather.
	1、little; 2、few; 3、less.
**10**	In the future ____ to get a lot of power from the sun.
	1、We'll need; 2、we are needing; 3、we can need.
**11**	Pele is still ____ famous footballer in the world.
	1、the most; 2、most; 3、 the more.
**12**	He ____ born in 1940.
	1、had been; 2、is; 3、was.
**13**	His mother _______ him to be a footballer.
	1、not want; 2、wasn't wanting; 3、didn't want.
**14**	But he _____ to watch his father play.
	1、used; 2、ought; 3、 has used.
**15**	His father ___ practice everyday.
	1、 made him to; 2、made him; 3、 would make him to.
**16**	He learned to use ________ and his right.
	1、or his left foot or; 2、and his left foot and; 3、both his left foot.
**17**	He got the name Pele when he ________.
	1、had only 10 years; 2、was only 10; 3、was only 10 years.
**18**	By 1956 he ______ Santos and had scored in his first game.
	1、 joined; 2、had joined; 3、 has joined.
**19**	In 1957 he ___ for the Brazilian national team.
	1、has been picked; 2、was picked; 3、 was picking.
**20**	The World Cup Finals were in 1958 and Pele was looking forward to _____.
	1、playing; 2、to play; 3、to be playing.
**21**	But he hurt ___ knee in a game in Brazil.
	1、the; 2、his; 3、this.
**22**	He thought he _________ be able to play in the finals in Sweden.
	1、isn't going to; 2、couldn't; 3、wasn't going to.
**23**	If he _______ so important to the team, he would have been left behind.
	1、hadn't been; 2、weren't; 3、wouldn't be.
**24**	But he was ____ brilliant player, they took him anyway.
	1、a such; 2、such a; 3、a so.
**25**	And _________ he was injured, he helped Brazil to win the final.
	1、even though; 2、even so; 3、 in spite of.

**Instruction 2:** Please tick the corresponding number you think is the correct answer in the table after the passage.

The history of the World Cup is 26 one. Football ____27____ played for ___28_____ a hundred years, but the first World Cup competition ___29______ held until 1930. Uruguay ___30___ the Olympic football final in 1924 and 1928 and wanted __31____ World Champions for the third time. Four teams entered from Europe, but with __32___ success. It was the first time __33___ professional teams __34____ for a world title. It wasn't until 4 years ___35____ that a European team succeeded ___36__ for _37__ first time. The 1934 World Cup was again won by_38______ home team, ____39__ has been the case several times since then. The 1934 final was __40_ two European teams, Czechoslovakia and Italy, _41___ won, went on ___42__ the 1938 final. Winning successive finals is something that ______43_ achieved again until Brazil did _44____ in 1958 and 1962. If Brazil ______45____ in 1966, then the Authorities would have needed to ___46__ the original World Cup replaced. But England stopped the Brazilians __47___ a successive win. An England player, Geoff Hurst, scored three goals in the final and won it almost ____48_____. 1966 proved _49_______ the last year that England ____50__ even qualify for the finals till 1982, though they got in as winners in 1970.

**Table:**

**26**	1、 quite a 2、 a quite 3、 quite
**27**	1、 has been 2、 is being 3、 was
**28**	1、 above 2、 over 3、 more that
**29**	1、 wasn't 2、 didn't be 3、 was not being
**30**	1、 could win 2、 were winning 3、 had won
**31**	1、 be 2、 being 3、 to be
**32**	1、 a little 2、 few 3、 little
**33**	1、 which 2、that 3、 when
**34**	1、 are playing 2、 would play 3、 had played
**35**	1、 later 2、 more 3、 further
**36**	1、 to win 2、 in winning 3、 at winning
**37**	1、the 2、a 3、 its
**38**	1、 a 2、 the 3、 one
**39**	1、 what 2、 this 3、 which
**40**	1、 among 2、 between 3、 against
**41**	1、 which 2、that 2、 who
**42**	1、 to win 2、 winning 3、 to have won
**43**	1、 is not 2、 was not 3、 has not been
**44**	1、 them 2、 these 3、 it
**45**	1、 would have won 2、 would win 3、 had won
**46**	1、 have 2、 let 3、 make
**47**	1、 to get 2、 getting 3、 get
**48**	1、 by his own 2、 on himself. 3、 by himself
**49**	1、 being 2、 as being 3、 to be
**50**	1、 would 2、 will 3、 did

## Appendix B. STIMULUS WORD LIST

The first 24 elements were practice items, the following 96 words were experimental items.

**Table:**

Chinese	English	Chinese	English	Chinese	English
Warm‐up words					
飞机	Airplane	蚂蚁	Ant	手臂	Arm
篮子	Basket	黑熊	Bear	蜜蜂	Bee
铃铛	Bell	大象	Elephant	青蛙	Frog
手指	Finger	旗帜	Flag	手枪	Gun
Formal words					
苹果	Apple	书包	Bag	皮球	Ball
香蕉	Banana	床铺	Bed	小鸟	Bird
小船	Boat	书本	Book	盒子	Box
面包	Bread	公交	Bus	蛋糕	Cake
汽车	Car	小猫	Cat	椅子	Chair
钟表	Clock	云朵	Cloud	外套	Coat
课桌	Desk	小狗	Dog	大门	Door
裙子	Dress	耳朵	Ear	眼睛	Eye
花朵	Flower	女孩	Girl	杯子	Glass
头发	Hair	帽子	Hat	房子	House
钥匙	Key	刀子	Knife	镜子	Mirror
猴子	Monkey	月亮	Moon	老鼠	Mouse
鼻子	Nose	铅笔	Pencil	电话	Phone
钢琴	Piano	小猪	Pig	玫瑰	Rose
衬衫	Shirt	星星	Star	火车	Train
大树	Tree	手表	Watch	窗户	Window

## Appendix C. NAMING BEHAVIORAL RESULTS

Naming latencies were skewed, so we first log transformed them before analyzing them with the linear mixed‐effect models in R using the lme4 package (Bates, Maechler, Bolker, & Walker, 2015) and lmerTest package (Kuznetsova, Brockhoff, & Christensen, 2017). The “Model <‐lmer(RT ~ Modality*Modality sequence*Language*language sequence + (1|Subject),data)” converged, and parameters were estimated with Restricted Maximum Likelihood (REML).

Tables C1, C2, C3, C4

**TABLE C1:** Main and interaction effects of naming latencies

Effects	Comparisons	*β*	*SE*	*t*	*p*
Modality sequence	Cross > Within	8.382e−02	1.348e−02	6.22	4.00e−09***
Language	L1 > L2	−5.875e−02	1.036e−02	−5.67	1.47e−08***
Language sequence	Switch > Repeat	6.306e−02	1.087e−02	5.80	1.53e−08***
Language sequence × Modality sequence	—	−6.706e−02	1.602e−02	−4.19	2.87e−05***

*Note*. Fixed‐effect predictors: Modality sequence (Cross .5 and Within −0.5), Language (L1 0.5 and L2–0.5), Language sequence (Switch 0.5 and Repeat −0.5). For each predictor, the estimate, standard error, *t* values, and *p* values are given. Asterisks indicate a significant effect.

**TABLE C2:** Main effects on regions of interest

Effects	ROIs	Comparisons	*β*	*SE*	*t*	*p*
Modality	L DLPFC	Production > Comprehension	15.32	1.93	7.93	<.0001***
	ACC	Production > Comprehension	18.14	1.82	10.00	<.0001***
	L Precuneus	Production > Comprehension	7.49	2.04	3.67	<.001***
Modality sequence	L DLPFC	Cross > Within	2.57	.70	3.68	<.001***
	ACC	Cross > Within	2.08	.56	3.74	<.001***
	L Precuneus	Cross > Within	3.36	.68	4.92	<.0001***
Language sequence	L Precuneus	Switch > Repeat	1.36	.66	2.05	.045*

*Note*. Fixed‐effect predictors: Modality (Production .5 and Comprehension −.5), Modality sequence (Cross .5 and Within −.5), Language (L1 .5 and L2–.5), Language sequence (Switch .5 and Repeat −.5). For each predictor, the estimate, standard error, *t* values, and *p* values are given. Asterisks indicate a significant effect.

Abbreviations: L, left; R, right.

**TABLE C3:** Interaction effects on regions of interest

Interaction effects	ROIs	*β*	*SE*	*t*	*p*
Modality × Modality sequence	L DLPFC	23.82	1.28	18.60	<.0001***
	ACC	23.88	1.11	21.50	<.0001***
	L Precuneus	13.51	1.20	11.24	<.0001***
Modality sequence × Language	L Precuneus	3.26	1.20	2.71	.007**
Modality × Modality sequence × Language sequence	L Precuneus	4.84	2.40	2.01	.045*

*Note*. Fixed‐effect predictors: Modality (Comprehension .5 and Production −.5), Modality sequence (Cross .5 and Within −.5), Language (L1 .5 and L2–.5), Language sequence (Switch .5 and Repeat −.5). For each predictor, the estimate, standard error, *t* values, and *p* values are given. Asterisks indicate a significant effect.

Abbreviations: L, left; R, right.

**TABLE C4:** Follow‐up analysis on interaction effects

Interaction effects	ROIs	Comparisons	*β*	*SE*	*t*	*p*
Modality × Modality sequence	L DLPFC	Within: Production > Comprehension	3.41	2.04	1.68	.121
		Cross: Production > Comprehension	27.23	2.04	13.38	<.0001***
		Production: Cross > Within	14.48	.95	15.28	<.0001***
		Comprehension: Within > Cross	−9.34	.95	−9.86	<.0001***
	ACC	Within: Production > Comprehension	6.20	1.90	3.27	.0024*
		Cross: Production > Comprehension	30.10	1.90	15.85	<.0001***
		Production: Cross > Within	14.02	.79	17.85	<.0001***
		Comprehension: Within > Cross	−9.86	.79	−12.55	<.0001***
	L Precuneus	Within: Production > Comprehension	.73	2.12	.34	.733
		Cross: Production > Comprehension	14.24	2.12	6.70	<.0001***
		Production: Cross > Within	10.1	.91	11.13	<.0001***
		Comprehension: Within > Cross	−3.4	.91	−3.74	.0003**
Modality sequence × Language	L Precuneus	L1: Cross > Within	4.99	.91	5.49	<.0001***
		L2: Cross > Within	1.73	.91	1.91	.059
		Within‐modality: L2 > L1	−.61	.85	−0.72	.471
		Cross‐modality: L1 > L2	2.64	.85	3.11	.002*
Modality × Modality sequence × Language sequence	L Precuneus	Within‐production: Switch > Repeat	2.55	1.23	2.07	.040*
		Cross‐production: Repeat > Switch	−1.57	1.23	−1.27	.204
		Within‐comprehension: Switch > Repeat	1.87	1.23	1.52	.130
		Cross‐comprehension: Switch > Repeat	2.59	1.23	2.09	.037*

Abbreviations: L, left; R, right.

As shown in Table C1, we obtained the same results as in the ANOVA. Follow‐up analyses for the interaction between Modality sequence × Language sequence showed that, in the Within‐modality condition, Switch trials were significantly slower than Repeat trials (*β* = −.06, *SE* = .01, *z* = −7.00, *p* < .0001), while such difference in the Cross‐modality condition was not significant (*β* = .003, *SE* = .01, *z* = 0.29, *p* = .772).

As shown in Table C1, we obtained the same results as in the ANOVA. Follow‐up analysis for the interaction between Modality sequence × Language sequence showed that, in the Within‐modality condition, Switch trials were significantly slower than Repeat trials (*β* = 51.92, *SE* = 7.34, *t* = 7.07, *p* < .0001), while such difference in the Cross‐modality condition was not significant (*β* = 1.38, *SE* = 7.38, *t* = .19, *p* = .852).

*ROI results*

The ROI statistical analysis were the same as the behavioral analysis in the linear mixed‐effect models.

Model_1 = lmer (ACC ~ Modality * Modality sequence * Language * Language sequence + (1 + Modality + Language sequence|Subject), data)

Model_2 = lmer (DLPFC ~ Modality * Modality sequence * Language * Language sequence + (1 + Modality + Modality sequence|Subject), data)

Model_3 <‐ lmer (Precuneus ~ Modality* Modality sequence * Language * Language sequence + (1 + Modality + Person sequence + Language sequence|Subject), data)

As shown in Table C2, we almost obtained the same main effects as in the ANOVA.

As shown in Tables C3 and C4, we almost obtained the same results as in the ANOVA. Further, we conducted follow‐up analyses for the interaction between Modality sequence × Language sequence in Production trials and in Comprehension trials. During production in the Within‐modality condition, Switch trials were significantly higher than Repeat trials (*β* = −2.55, *SE* = 1.23, *t* = 2.07, *p* = .040), while no such difference was found in the Cross‐modality condition (*β* = 1.57, *SE* = 1.23, *t* = 1.28, *p* = .203). In comprehension, Switch trials were higher than Repeat trials in Cross‐modality condition (*β* = −2.59, *SE* = 1.22, *t* = −2.13, *p* = .034), but there was no significant difference between Switch and Repeat trials in the Within‐modality condition (*β* = −1.87, *SE* = 1.22, *t* = −1.54, *p* = .125). These findings were consistent with ANOVA results.

## References

[hbm25044-bib-0001] Abutalebi, J. , Annoni, J. M. , Zimine, I. , Pegna, A. J. , Seghier, M. L. , Lee‐Jahnke, H. , … Khateb, A. (2008). Language control and lexical competition in bilinguals: An event‐related fMRI study. Cerebral Cortex, 18(7), 1496–1505. 17947346 10.1093/cercor/bhm182

[hbm25044-bib-0002] Abutalebi, J. , Della Rosa, P. A. , Ding, G. , Weekes, B. , Costa, A. , & Green, D. W. (2013). Language proficiency modulates the engagement of cognitive control areas in multilinguals. Cortex, 49(3), 905–911. 23021069 10.1016/j.cortex.2012.08.018

[hbm25044-bib-0003] Abutalebi, J. , & Green, D. W. (2016). Neuroimaging of language control in bilinguals: Neural adaptation and reserve. Bilingualism: Language and Cognition, 19(4), 689–698.

[hbm25044-bib-0004] Allan, D. (2004). Oxford placement test 2: Test pack, Oxford, England: Oxford University Press.

[hbm25044-bib-0065] Bates, D. , Mächler, M. , Bolker, B. , & Walker, S. (2015). Fitting linear mixed‐effects models using lme4. Journal of Statistical Software, 67(1), 133–199.

[hbm25044-bib-0005] Baus, C. , Sebanz, N. , Fuente, V. D. L. , Branzi, F. M. , Martin, C. , & Costa, A. (2014). On predicting others' words: Electrophysiological evidence of prediction in speech production. Cognition, 133(2), 395–407. 25128797 10.1016/j.cognition.2014.07.006

[hbm25044-bib-0006] Bischoff‐Grethe, A. , Ivry, R. B. , & Grafton, S. T. (2002). Cerebellar involvement in response reassignment rather than attention. Journal of Neuroscience, 22(2), 546–553. 11784801 10.1523/JNEUROSCI.22-02-00546.2002 PMC6758676

[hbm25044-bib-0007] Blanco‐Elorrieta, E. , Emmorey, K. , & Pylkkanen, L. (2018). Language switching decomposed through MEG and evidence from bimodal bilinguals. Proceedings of the National Academy of Sciences, 115(39), 9708–9713. 10.1073/pnas.1809779115 PMC6166835 30206151

[hbm25044-bib-0008] Blanco‐Elorrieta, E. , & Pylkkänen, L. (2016). Bilingual language control in perception versus action: MEG reveals comprehension control mechanisms in anterior cingulate cortex and domain‐general control of production in dorsolateral prefrontal cortex. Journal of Neuroscience, 36(2), 290–301. 26758823 10.1523/JNEUROSCI.2597-15.2016 PMC6602022

[hbm25044-bib-0009] Blanco‐Elorrieta, E. , & Pylkkänen, L. (2017). Bilingual language switching in the lab vs. in the wild: The spatio‐temporal dynamics of adaptive language control. Journal of Neuroscience, 37, 9022–9036. 28821648 10.1523/JNEUROSCI.0553-17.2017 PMC5597983

[hbm25044-bib-0010] Branzi, F. M. , Della Rosa, P. A. , Canini, M. , Costa, A. , & Abutalebi, J. (2015). Language control in bilinguals: Monitoring and response selection. Cerebral Cortex, 26(6), 2367–2380. 25838037 10.1093/cercor/bhv052

[hbm25044-bib-0011] Brysbaert, M. , & New, B. (2009). Moving beyond Kučera and Francis: A critical evaluation of current word frequency norms and the introduction of a new and improved word frequency measure for American English. Behavior Research Methods, 41(4), 977–990. 19897807 10.3758/BRM.41.4.977

[hbm25044-bib-0012] Cai, Q. , & Brysbaert, M. (2010). SUBTLEX‐CH: Chinese word and character frequencies based on film subtitles. PLoS One, 5(6), e10729. 20532192 10.1371/journal.pone.0010729 PMC2880003

[hbm25044-bib-0013] Cavanna, A. E. , & Trimble, M. R. (2006). The precuneus: A review of its functional anatomy and behavioural correlates. Brain, 129(3), 564–583. 16399806 10.1093/brain/awl004

[hbm25044-bib-0014] Chikazoe, J. , Konishi, S. , Asari, T. , Jimura, K. , & Miyashita, Y. (2007). Activation of right inferior frontal gyrus during response inhibition across response modalities. Journal of Cognitive Neuroscience, 19(1), 69–80. 17214564 10.1162/jocn.2007.19.1.69

[hbm25044-bib-0015] Costa, A. , & Santesteban, M. (2004). Lexical access in bilingual speech production: Evidence from language switching in highly proficient bilinguals and L2 learners. Journal of Memory and Language, 50(4), 491–511.

[hbm25044-bib-0016] Costa, A. , Santesteban, M. , & Ivanova, I. (2006). How do highly proficient bilinguals control their lexicalization process? Inhibitory and language‐specific selection mechanisms are both functional. Journal of Experimental Psychology: Learning, Memory & Cognition, 32, 1057–1074. 10.1037/0278-7393.32.5.1057 16938046

[hbm25044-bib-0017] de Bruin, A. , Roelofs, A. , Dijkstra, T. , & FitzPatrick, I. (2014). Domain‐general inhibition areas of the brain are involved in language switching: fMRI evidence from trilingual speakers. NeuroImage, 90, 348–359. 24384153 10.1016/j.neuroimage.2013.12.049

[hbm25044-bib-0018] Declerck, M. , Koch, I. , Duñabeitia, J. A. , Grainger, J. , & Stephan, D. N. (2019). What absent switch costs and mixing costs during bilingual language comprehension can tell us about language control. Journal of Experimental Psychology: Human Perception and Performance, 45(6), 771–789. 30920253 10.1037/xhp0000627

[hbm25044-bib-0019] Declerck, M. , & Philipp, A. M. (2015). A review of control processes and their locus in language switching. Psychonomic Bulletin & Review, 22(6), 1630–1645. 25917142 10.3758/s13423-015-0836-1

[hbm25044-bib-0020] Dijkstra, A. (. T.). , & van Heuven, W. J. B. (1998). The BIA model and bilingual word recognition In Grainger J. & Jacobs A. M. (Eds.), Localist connectionist approaches to human cognition (pp. 189–225). Mahwah, NJ: Lawrence Erlbaum Associates.

[hbm25044-bib-0021] Dijkstra, T. , & van Heuven, W. J. B. (2002). The architecture of the bilingual word recognition system: From identification to decision. Bilingualism: Language and Cognition, 5(3), 175–197.

[hbm25044-bib-0022] Dosenbach, N. U. , Fair, D. A. , Miezin, F. M. , Cohen, A. L. , Wenger, K. K. , Dosenbach, R. A. , … Schlaggar, B. L. (2007). Distinct brain networks for adaptive and stable task control in humans. Proceedings of the National Academy of Sciences, 104(26), 11073–11078. 10.1073/pnas.0704320104 PMC1904171 17576922

[hbm25044-bib-0023] Farrer, C. , & Frith, C. D. (2002). Experiencing oneself vs another person as being the cause of an action: The neural correlates of the experience of agency. NeuroImage, 15(3), 596–603. 11848702 10.1006/nimg.2001.1009

[hbm25044-bib-0024] Frings, C. , Hommel, B. , Koch, I. , Rothermund, K. , Dignath, D. , Giesen, C. , … Philipp, A. (2020). Binding and retrieval in action control (BRAC). Trends in Cognitive Sciences, 24, 375–387. 10.1016/j.tics.2020.02.004 32298623

[hbm25044-bib-0025] Gambi, C. , & Hartsuiker, R. J. (2016). If you stay, it might be easier: Switch costs from comprehension to production in a joint switching task. Journal of Experimental Psychology: Learning, Memory & Cognition, 42(4), 608–626. 10.1037/xlm0000190 26461033

[hbm25044-bib-0028] Giezen, M. R. , Blumenfeld, H. K. , Shook, A. , Marian, V. , & Emmorey, K. (2015). Parallel language activation and inhibitory control in bimodal bilinguals. Cognition, 141, 9–25. 25912892 10.1016/j.cognition.2015.04.009 PMC4466161

[hbm25044-bib-0029] Grainger, J. , & Dijkstra, T. (1992). On the representation and use of language information in bilinguals In Advances in psychology (Vol. 83, pp. 207–220). North‐Holland: Elsevier.

[hbm25044-bib-0030] Grainger, J. , Midgley, K. , & Holcomb, P. J. (2010). Chapter 14. Re‐thinking the bilingual interactive‐activation model from a developmental perspective (BIA‐d) In Kail M. (Ed.), Language acquisition across linguistic and cognitive systems (pp. 267–283). New York, NY: John Benjamins Publishing Company.

[hbm25044-bib-0031] Green, D. W. (1998). Mental control of the bilingual lexico‐semantic system. Bilingualism: Language and Cognition, 1, 67–81.

[hbm25044-bib-0032] Guo, T. , Liu, H. , Misra, M. , & Kroll, J. F. (2011). Local and global inhibition in bilingual word production: fMRI evidence from Chinese–English bilinguals. NeuroImage, 56(4), 2300–2309. 21440072 10.1016/j.neuroimage.2011.03.049 PMC3741343

[hbm25044-bib-0033] Henson, R. N. , Eckstein, D. , Waszak, F. , Frings, C. , & Horner, A. J. (2014). Stimulus–response bindings in priming. Trends in Cognitive Sciences, 18(7), 376–384. 24768034 10.1016/j.tics.2014.03.004 PMC4074350

[hbm25044-bib-0034] Hernandez, A. E. (2009). Language switching in the bilingual brain: what's next? Brain and Language, 109(2), 133–140. 19250662 10.1016/j.bandl.2008.12.005

[hbm25044-bib-0035] Hernandez, A. E. , Martinez, A. , & Kohnert, K. (2000). In search of the language switch: An fMRI study of picture naming in Spanish‐English bilinguals. Brain and Language, 73(3), 421–431. 10860563 10.1006/brln.1999.2278

[hbm25044-bib-0036] Kootstra, G. J. , Hell, J. G. V. , & Dijkstra, T. (2010). Syntactic alignment and shared word order in code‐switched sentence production: Evidence from bilingual monologue and dialogue. Journal of Memory and Language, 63(2), 210–231.

[hbm25044-bib-0066] Kuznetsova, A. , Brockhoff, P. B. , & Christensen, R. H. B. (2017). lmerTest package: Tests in linear mixed effects models. Journal of Statistical Software, 82(13), 1–26.

[hbm25044-bib-0037] Li, B. , Liu, H. , Pérez, A. , & Xie, N. (2018). Cathodal transcranial direct current stimulation over right dorsolateral prefrontal cortex improves language control during language switching. Behavioural Brain Research, 351, 34–41. 29842915 10.1016/j.bbr.2018.05.026

[hbm25044-bib-0038] Liang, L. , & Chen, B. (2014). Processing morphologically complex words in second‐language learners: The effect of proficiency. Acta Psychologica, 150, 69–79. 24824457 10.1016/j.actpsy.2014.04.009

[hbm25044-bib-0039] Liu, H. , Liang, L. , Dunlap, S. , Fan, N. , & Chen, B. (2016). The effect of domain‐general inhibition‐related training on language switching: An ERP study. Cognition, 146, 264–276. 26491833 10.1016/j.cognition.2015.10.004

[hbm25044-bib-0040] Loose, R. , Kaufmann, C. , Auer, D. P. , & Lange, K. W. (2003). Human prefrontal and sensory cortical activity during divided attention tasks. Human Brain Mapping, 18(4), 249–259. 12632463 10.1002/hbm.10082 PMC6871829

[hbm25044-bib-0041] Luk, G. , Green, D. W. , Abutalebi, J. , & Grady, C. (2012). Cognitive control for language switching in bilinguals: A quantitative meta‐analysis of functional neuroimaging studies. Language & Cognitive Processes, 27(10), 1479–1488. 10.1080/01690965.2011.613209 PMC4006828 24795491

[hbm25044-bib-0042] MacDonald, A. W. , Cohen, J. D. , Stenger, V. A. , & Carter, C. S. (2000). Dissociating the role of the dorsolateral prefrontal and anterior cingulate cortex in cognitive control. Science, 288(5472), 1835–1838. 10846167 10.1126/science.288.5472.1835

[hbm25044-bib-0043] Meuter, R. F. , & Allport, A. (1999). Bilingual language switching in naming: Asymmetrical costs of language selection. Journal of Memory and Language, 40(1), 25–40.

[hbm25044-bib-0044] Northoff, G. , & Bermpohl, F. (2004). Cortical midline structures and the self. Trends in Cognitive Sciences, 8(3), 102–107. 15301749 10.1016/j.tics.2004.01.004

[hbm25044-bib-0045] Novick, J. M. , Kan, I. P. , Trueswell, J. C. , & Thompson‐Schill, S. L. (2009). A case for conflict across multiple domains: Memory and language impairments following damage to ventrolateral prefrontal cortex. Cognitive Neuropsychology, 26(6), 527–567. 20183014 10.1080/02643290903519367 PMC3791076

[hbm25044-bib-0046] Novick, J. M. , Trueswell, J. C. , & Thompson‐Schill, S. L. (2005). Cognitive control and parsing: Reexamining the role of Broca's area in sentence comprehension. Cognitive, Affective, & Behavioral Neuroscience, 5(3), 263–281. 10.3758/cabn.5.3.263 16396089

[hbm25044-bib-0047] Peeters, D. , Runnqvist, E. , Bertrand, D. , & Grainger, J. (2014). Asymmetrical switch costs in bilingual language production induced by reading words. Journal of Experimental Psychology: Learning, Memory, and Cognition, 40(1), 284. 10.1037/a0034060 23957363

[hbm25044-bib-0048] Petrini, K. , Piwek, L. , Crabbe, F. , Pollick, F. E. , & Garrod, S. (2015). Look at those two!: The precuneus role in unattended third‐person perspective of social interactions. Human Brain Mapping, 35(10), 5190–5203. 10.1002/hbm.22543 PMC6869405 24824165

[hbm25044-bib-0049] Philipp, A. M. , Gade, M. , & Koch, I. (2007). Inhibitory processes in language switching: Evidence from switching language‐defined response sets. European Journal of Cognitive Psychology, 19(3), 395–416.

[hbm25044-bib-0050] Philipp, A. M. , & Koch, I. (2009). Inhibition in language switching: What is inhibited when switching between languages in naming tasks? Journal of Experimental Psychology: Learning, Memory, and Cognition, 35(5), 1187. 10.1037/a0016376 19686014

[hbm25044-bib-0052] Reverberi, C. , Kuhlen, A. K. , Abutalebi, J. , Greulich, R. S. , Costa, A. , Seyed‐Allaei, S. , & Haynes, J. D. (2015). Language control in bilinguals: Intention to speak vs. execution of speech. Brain and Language, 144, 1–9. 25868150 10.1016/j.bandl.2015.03.004

[hbm25044-bib-0053] Reverberi, C. , Kuhlen, A. K. , Seyed‐Allaei, S. , Greulich, R. S. , Costa, A. , Abutalebi, J. , & Haynes, J. D. (2018). The neural basis of free language choice in bilingual speakers: Disentangling language choice and language execution. NeuroImage, 177, 108–116. 29753107 10.1016/j.neuroimage.2018.05.025

[hbm25044-bib-0054] Seo, R. , Stocco, A. , & Prat, C. S. (2018). The bilingual language network: Differential involvement of anterior cingulate, basal ganglia and prefrontal cortex in preparation, monitoring, and execution. NeuroImage, 174, 44–56. 29486320 10.1016/j.neuroimage.2018.02.010

[hbm25044-bib-0055] Shomstein, S. , & Behrmann, M. (2006). Cortical systems mediating visual attention to both objects and spatial locations. Proceedings of the National Academy of Sciences, 103(30), 11387–11392. 10.1073/pnas.0601813103 PMC1544095 16840559

[hbm25044-bib-0056] Sperduti, M. , Delaveau, P. , Fossati, P. , & Nadel, J. (2011). Different brain structures related to self‐ and external‐agency attribution: A brief review and meta‐analysis. Brain Structure & Function, 216(2), 151–157. 21212978 10.1007/s00429-010-0298-1

[hbm25044-bib-0057] Starreveld, P. A. , De Groot, A. M. , Rossmark, B. M. , & Van Hell, J. G. (2014). Parallel language activation during word processing in bilinguals: Evidence from word production in sentence context. Bilingualism: Language and Cognition, 17(2), 258–276.

[hbm25044-bib-0067] Stasenko, A. , Hays, C. , Wierenga, C. E. , & Gollan, T. H. (2020). Cognitive control regions are recruited in bilinguals’ silent reading of mixed‐language paragraphs. Brain and Language, 204 10.1016/j.bandl.2020.104754. PMC7205452 32113072

[hbm25044-bib-0058] Utevsky, A. V. , Smith, D. V. , & Huettel, S. A. (2014). Precuneus is a functional core of the default‐mode network. Journal of Neuroscience, 34(3), 932–940. 24431451 10.1523/JNEUROSCI.4227-13.2014 PMC3891968

[hbm25044-bib-0059] van Heuven, W. J. , Dijkstra, T. , & Grainger, J. (1998). Orthographic neighborhood effects in bilingual word recognition. Journal of Memory and Language, 39(3), 458–483.

[hbm25044-bib-0060] van Heuven, W. J. , Schriefers, H. , Dijkstra, T. , & Hagoort, P. (2008). Language conflict in the bilingual brain. Cerebral Cortex, 18(11), 2706–2716. 18424776 10.1093/cercor/bhn030 PMC2567421

[hbm25044-bib-0061] Waszak, F. , Hommel, B. , & Allport, A. (2003). Task‐switching and long‐term priming: Role of episodic stimulus–task bindings in task‐shift costs. Cognitive Psychology, 46(4), 361–413. 12809680 10.1016/s0010-0285(02)00520-0

[hbm25044-bib-0062] Wu, J. , Yang, J. , Chen, M. , Li, S. , Zhang, Z. , Kang, C. , … Guo, T. (2019). Brain network reconfiguration for language and domain‐general cognitive control in bilinguals. NeuroImage, 199, 454–465. 31200066 10.1016/j.neuroimage.2019.06.022

[hbm25044-bib-0063] Yan, C. , Wang, X. , Zuo, X. , & Zang, Y. (2016). DPABI: Data Processing & Analysis for (resting‐state) brain imaging. Neuroinformatics, 14(3), 339–351. 27075850 10.1007/s12021-016-9299-4

[hbm25044-bib-0064] Zhang, Q. , & Yang, Y. (2003). The determiners of picture naming latency. Acta Psychologica Sinica, 35(04), 447–454.

